# Supplementary material for: Screening and Characterization of a New Iflavirus Virus in the Fruit Tree Pest Pyrops candelaria
Source: Insects. 2024 Aug 19;15(8):625. doi: 10.3390/insects15080625 (PMC11354621; doi:10.3390/insects15080625)
Supplement: Supplementary file 1 [file insects-15-00625-s001.zip › Supplementary Figure 1.pdf]

GGTCTATGCTGTATCCAAATTAAAGTATTTTTGCATGCAGGAAAAACTAATCTAAAGCGT
AAGCCACCGCGAAAATAACGAAAGCCAGAAAGAAGCAATGGGATAACACCGTCTCTAC
ATCTATTAGCAGTATAATTTATAACGCCATTATTTTCATACGCATCATGACCAATACCTT
GTGGATTACAAGTAAACATAAAAGCACACTTATCATAATTAGAAAGCAAAGCAGGATTA
ATAGTAACTCTACAATATAATTGATATCGTCTACATAAAGTCAATAAATCATTAAAATTT
TCACCAAAATCTATATGTCCATATTGAGTTGTCTTTAACGATCTTCCCATAGGCAATACC
TCAACATCCGTACCACCAGGTTCTTCTCGCTCACCAGCTTGACAATAT

Figure S1. The length of the amplified DNA fragment by the diagnostic PCR is 405 bp.
