# Supplementary material for: Screening and Characterization of a New Iflavirus Virus in the Fruit Tree Pest Pyrops candelaria
Source: Insects. 2024 Aug 19;15(8):625. doi: 10.3390/insects15080625 (PMC11354621; doi:10.3390/insects15080625)
Supplement: Supplementary file 1 [file insects-15-00625-s001.zip › Supplementary Figure 2.pdf]

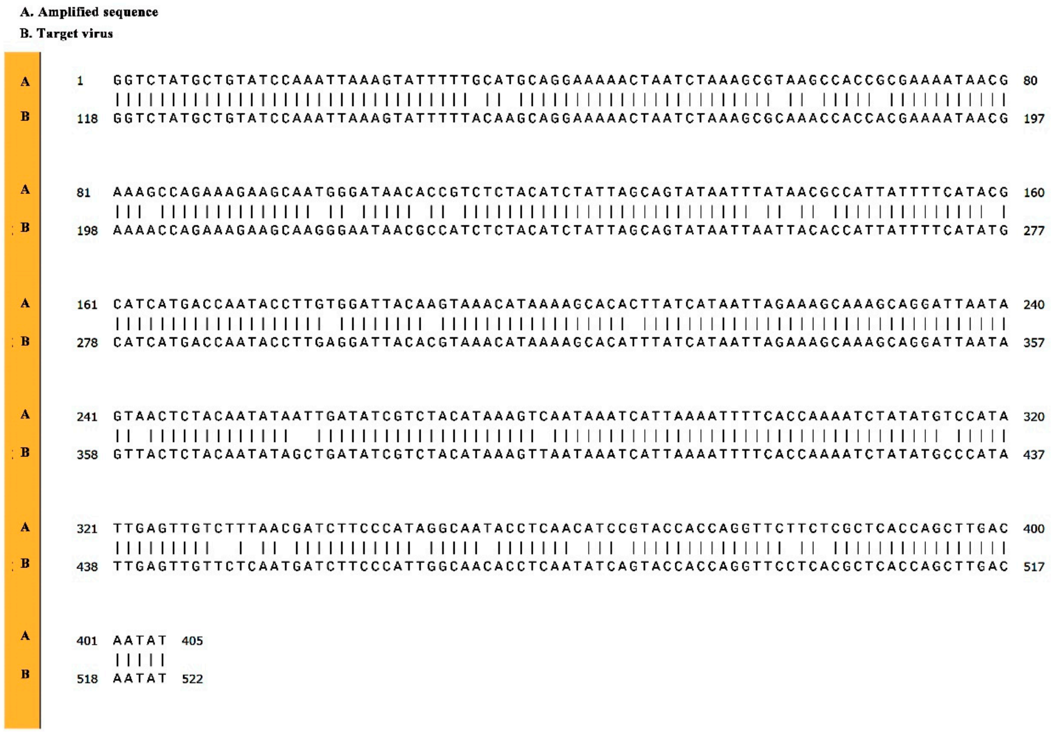

Figure S2. The alignment of target virus fragment and the amplified sequence. Note that A indicates the length of the amplified DNA fragment by the diagnostic PCR and B indicates the target virus fragment.
