# Supplementary material for: Screening and Characterization of a New Iflavirus Virus in the Fruit Tree Pest Pyrops candelaria
Source: Insects. 2024 Aug 19;15(8):625. doi: 10.3390/insects15080625 (PMC11354621; doi:10.3390/insects15080625)
Supplement: Supplementary file 1 [file insects-15-00625-s001.zip › Supplementary Table 2_primers of RACE.pdf]

Table S2 The primers of RACE experiment.

Primer name	Primer sequences	Description
FW5052-5YZ-F1	GTAGAGGGCTGGGATCATGGTAC	
FW5052-5YZ-F2	GGATGGATTTACAGGTCCATTGAC	
FW5052-5YZ-R1	CGAAAAGCAGGATGATCATCAAA	
FW5052-5YZ-R2	CGGAAAATCTTCAATATTAAGAGGAGC	Target gene homologous amplification primers
FW5052-3YZ-F1	CTGTCCCCTGGTGTGAATTAGATAG	
FW5052-3YZ-F2	AAGGGGACTATAAACGTAGAGTTAATGC	
FW5052-3YZ-R1	AAGAAACAGTAAATATGCGCCACTC	
FW5052-3YZ-R2	AGAGCGATACGCCTGAAGTAGAC	
FW5052- 3RACE-GSP1	CTGTCCCCTGGTGTGAATTAGATAG	Primers for 3 RACE amplification of target gene
FW5052- 5RACE-GSP1	GGGTCAATACTACCAATAGGGAGGACT	5 ' RACE amplification primers of target gene
FW5052- 5RACE-GSP2	GATTACGCCAAGCTtGCAAGGTACACCTGAAGACGTGAGT	
FW5052-PF1	CGACGACTCATTGACAAGCATAG	
FW5052-PF2	GATCAGGATAATGACGCACGAAC	
FW5052-PF3	TTTGCCATCAGGGTCATATTGTC	
FW5052-PF4	GAACTTTTCATATGGTATTCGTGGTG	
FW5052-PF5	ACGCTTAATAGGTATTTGCTTGGTAGA	
FW5052-PF6	GAACGTGATCCGCTTACGCTT	Amplify the intermediate region of the target gene
FW5052-PR1	TTTTAGCTACATTTTCACCGACTGA	
FW5052-PR2	ATGATCTTCCCATTGGCAACAC	
FW5052-PR3	ATACATACACGCCAAATGGTTCC	
FW5052-PR4	GGCCAATCCCATAAACATACTCC	
FW5052-PR5	CCCGCAGAAGTAGTCCAAGCTA	
FW5052-PR6	CCTGAAGTAGACCCTTATCTAGCAGGT	
