# Supplementary material for: Screening and Characterization of a New Iflavirus Virus in the Fruit Tree Pest Pyrops candelaria
Source: Insects. 2024 Aug 19;15(8):625. doi: 10.3390/insects15080625 (PMC11354621; doi:10.3390/insects15080625)
Supplement: Supplementary file 1 [file insects-15-00625-s001.zip › Supplementary Table 3_ virus_abbre.pdf]

Table S3 The abbreviation of virus in the construction of phylogenetic tree in this study 1

**Description**	**Virus number**	**Virus abbreviations**
NP_277061.1:1-2986_polyprotein_organism_Perina_nuda_virus	NP_277061	PnIV
NP_620559.1:1-3085_polyprotein_organism_Infectious_flacherie_virus	NP_620559	IFV
NP_919029.1:1-2987_polyprotein_organism_Ectropis_obliqua_picorna-like_virus	NP_919029	EoV
YP_001285409.1:1-2983_polyprotein_organism_Brevicoryne_brassicae_virus_-_UK	Y1285409	BBV
YP_003622540.1:1-2964_polyprotein_organism_Slow_bee_paralysis_virus	Y3622540	SBPV
YP_008130309.1:1-3245_polyprotein_organism_Nilaparvata_lugens_honeydew_virus-2_isolate_Izumo	Y8130309	NLHV2
YP_008719809.1:1-3016_polyprotein_organism_Halyomorpha_halys_virus_isolate_Beltsville	Y8719809	HhV
YP_008888537.1:1-2910_polyprotein_organism_Formica_exsecta_virus_2_isolate_Fex2	Y8888537	Fex2
YP_009002581.1:1-3036_polyprotein_organism_Antheraea_pernyi_iflavirus_isolate_LnApIV-02	Y9002581	ApIV
YP_009010941.1:1-3125_polyprotein_organism_Laodelphax_striatella_honeydew_virus_1_isolate_Nanjing	Y9010941	LsHV1
YP_009010984.1:1-3010_polyprotein_organism_Spodoptera_exigua_iflavirus_2_isolate_Korean	Y9010984	SEIV2
YP_009026409.1:1-2965_polyprotein_organism_Heliconius_erato_iflavirus	Y9026409	HeIV
YP_009047245.1:1-2980_polyprotein_organism_Lymantria_dispar_iflavirus_1_isolate_Ames	Y9047245	LdIV1
YP_009110667.1:1-3125_polyprotein_organism_Laodelphax_striatellus_picorna-like_virus_2_isolate_LsPV2	Y9110667	LsPV2
YP_009111311.1:1-3006_polyprotein_organism_Dinocampus_coccinellae_paralysis_virus	Y9111311	DcPV
YP_009116875.1:1-2957_polyprotein_organism_Thaumetopoea_pityocampa_iflavirus_1	Y9116875	TpIV1
YP_009129265.1:1-3032_polyprotein_organism_Graminella_nigrifrons_virus_1_isolate_Ohio	Y9129265	GnV1
YP_009140562.1:1-3057_putative_polyprotein_organism_La_Jolla_virus_isolate_MAT03	Y9140562	LJV
YP_009162630.1:1-3004_polyprotein_organism_Bombyx_mori_iflavirus	Y9162630	BMIV
YP_009305421.1:1-3050_polyprotein_organism_Moku_virus_isolate_Big_Island	Y9305421	Moku_virus
YP_009328891.1:1-3132_polyprotein_organism_Euscelidius_variegatus_virus_1_isolate_to-1	Y9328891	Euscelidius_variegatus_virus
YP_009344960.1:1-3021_polyprotein_organism_Helicoverpa_armigera_iflavirus	Y9344960	Helicoverpa_armigera_iflavirus
YP_009345906.1:1-3179_polyprotein_organism_Bat_iflavirus	Y9345906	BatIV
YP_009351892.1:1-3057_polyprotein_organism_Pityohyphantes_rubrofasciatus_iflavirus_isolate_UW1	Y9351892	Pityohyphantes_rubrofasciatus_iflavirus
YP_009361829.1:1-3012_polyprotein_organism_Diamondback_moth_iflavirus_isolate_Guangzhou	Y9361829	Diamondback_moth_iflavirus

Table S4 The abbreviation of virus in the construction of phylogenetic tree in this study(continued)

**Description**	**Virus number**	**Virus abbreviations**
YP_009444707.1:1-2981_polyprotein_organism_Chequa_iflavirus_isolate_A14-49.4	Y9444707	Chequa_iflavirus
YP_009448183.1:1-2752_polyprotein_organism_Armigeres_iflavirus	Y9448183	Armigeres_iflavirus
YP_009505598.1:1-2986_polypeptide_organism_Lygus_lineolaris_virus_1_isolate_LlV-1	Y9505598	LyLV1
YP_009505599.1:1-3175_polyprotein_organism_Nilaparvata_lugens_honeydew_virus_1_isolate_Izumo	Y9505599	NLHV1
YP_009552017.1:1-3115_polyprotein_organism_Culex_Iflavi-like_virus_4	Y9552017	Culex_Iflavi_like_virus_4
YP_009552080.1:1-2761_polyprotein_organism_Yongsan_iflavirus_1	Y9552080	Yongsan_iflavirus_1
YP_009552119.1:1-2569_polyprotein_organism_Varroa_destructor_virus_2_isolate_VDV-2	Y9552119	VDV2
YP_009552768.1:1-3277_polyprotein_organism_Culex_Iflavi-like_virus_1	Y9552768	Culex_Iflavi_like_virus_1
YP_009553231.1:1-3130_polyprotein_organism_Culex_Iflavi-like_virus_4	Y9553231	Culex_Iflavi_like_virus_4
YP_009553259.1:1-3187_polyprotein_organism_Psammotettix_alienus_iflavirus_1	Y9553259	PaIV1
YP_009553638.1:1-3119_polyprotein_organism_Culex_Iflavi-like_virus_4	Y9553638	Culex_Iflavi_like_virus_4
YP_010840358.1:1-2817_polyprotein_organism_Acheta_domesticus_iflavirus_isolate_SE:1050	YP_010840358	AdIV2
YP_145791.1:1-2893_polyprotein_organism_Varroa_destructor_virus_1	YP_145791	VDV1
ORF1_RdIV1:1137:10724_unnamed_protein_product	ORF1_RdIV1	RdIV1
ORF1_MfIV1:1050:10415_unnamed_protein_product	ORF1_MfIV1	MfIV1
YP_009315906.1:1-3037_polyprotein_organism_King_virus	Y9315906	King_virus
YP_008130310.1:1-3175_polyprotein_organism_Nilaparvata_lugens_honeydew_virus-3_isolate_Kagoshima	Y8130310	NLHV3
YP_009165593.1:1-3185_polyprotein_organism_Opsiphanes_invirae_iflavirus_1_isolate_Brazilian/2012	Y9165593	OilV_1
NP_049374.1:1-2858_polyprotein_organism_Sacbrood_virus	NP_049374	SBV
NP_853560.2:1-2893_polyprotein_organism_Deformed_wing_virus	NP_853560	DWV
YP_004935363.1:1-3222_polyprotein_organism_Spodoptera_exigua_iflavirus_1	Y4935363	SEIV1
ORF1_TBD:1057:10572_unnamed_protein_product	ORF1_TBD	TBD
YP_010084736.1_polyprotein_Rabovirus_D1		rvd
YP_010084737.1_polyprotein_Aimelvirus_1		av1
