# Supplementary material for: Screening and Characterization of a New Iflavirus Virus in the Fruit Tree Pest Pyrops candelaria
Source: Insects. 2024 Aug 19;15(8):625. doi: 10.3390/insects15080625 (PMC11354621; doi:10.3390/insects15080625)
Supplement: Supplementary file 1 [file insects-15-00625-s001.zip › Supplementary Table 4_samples collected in different seasons and regions.pdf]

Table S4. The number of longan lanternflies and PyCaV carrying samples collected in different month and regions.

**Month**		**3**	**6**	**9**	**10**	**12**
**FRIFAAS**	**Total (positive)**	14（4）	13（1）	7（1）	17（4）	10（1）
	**Positive rate (%)**	28.6	7.7	14.3	23.5	10
**Minhou County**	**Total (positive)**	12（8）	13（3）	11（3）	10（3）	16（5）
	**Positive rate (%)**	66.7	23.1	27.3	30	31.3
