# Supplementary material for: Screening and Characterization of a New Iflavirus Virus in the Fruit Tree Pest Pyrops candelaria
Source: Insects. 2024 Aug 19;15(8):625. doi: 10.3390/insects15080625 (PMC11354621; doi:10.3390/insects15080625)
Supplement: Supplementary file 1 [file insects-15-00625-s001.zip › Supplementary Table 5_Reagents and equipment used in this experiment.pdf]

Table S5-1 Laboratory reagents and their manufacturers

**Reagent name**	**Reagent manufacturers**
Hieff High Fidelity DNA Polymerase	Yeasen, Shanghai, China
Trizol Total RNA Extraction Reagent	Batek, China
One-step gDNA Removal and cDNA Synthesis SuperMix	TransGen Biotech, China
Easy Taq DNA polymerase	Yeasen, Shanghai, China
Taq Pro Universal SYBR qPCR Master Mix	Vazyme, Nanjing, China

Table S5-2 Laboratory equipment and its manufacturers

**Instrument name**	**Company**
PCR instrument	Thermo ProFlexTM
Electrophoresis apparatus	Bio-red
Biosafety cabinet	Thermo
Refrigerated centrifuge	Eppendorff
Nanodrop 2000	Thermo
Gel imager	Vilbre, France
